# Blisters in Children

**Published:** 1845-04

**Authors:** 


*Blisters in Children.*---Some discussion took place respecting the use of blisters in children. Generally, their employment was looked upon as only a choice of evils, and two cases were related in which their application produced fatal results. The president had found, in cases where blistered surfaces were healed with difficulty, that the mixture of a grain or two of opium with an ounce of spermaceti ointment was of great benefit. In cases in which morphia was employed endermically, the difficulty often was, to keep the blistered surface open.---*Lon. Med. Soc. Rep. in Lancet.—Bost. Med. and Surg. Jour.*

